# Development of a barley reference material for gluten analysis

**DOI:** 10.1016/j.foodchem.2023.136414

**Published:** 2023-10-30

**Authors:** Majlinda Xhaferaj, Gabriella Muskovics, Eszter Schall, Zsuzsanna Bugyi, Sándor Tömösközi, Katharina A. Scherf

**Affiliations:** a Karlsruhe Institute of Technology (KIT), Institute of Applied Biosciences, Department of Bioactive and Functional Food Chemistry, Karlsruhe, Germany; b Budapest University of Technology and Economics, Department of Applied Biotechnology and Food Science, Research Group of Cereal Science and Food Quality, Budapest, Hungary

**Keywords:** Celiac disease, Enzyme-linked immunosorbent assay (ELISA), Hordeins, Reversed-phase high-performance liquid chromatography (RP-HPLC), Sodium dodecyl sulfate polyacrylamide gel electrophoresis (SDS-PAGE), Wheat allergy

## Abstract

Celiac disease (CD) can be triggered in susceptible individuals by the consumption of gluten, a complex storage protein mixture present in wheat, rye and barley. There is no specific reference material (RM) available for barley and this leads to inaccurate quantitation of barley gluten in supposedly gluten-free foods. Therefore, the aim was to select representative barley cultivars to establish a new barley RM. The relative protein composition of the 35 barley cultivars averaged 25% albumins and globulins, 11% d-hordeins, 19% C-hordeins, and 45% B/γ-hordeins. The mean gluten and protein content was 7.2 g/100 g and 11.2 g/100 g, respectively. The prolamin/glutelin ratio (1:1) commonly used in ELISAs to calculate the gluten content was found to be inappropriate for barley (1.6 ± 0.6). Eight cultivars suitable as potential RMs were selected to ensure a typical barley protein composition and improve food safety for CD patients.

## Highlights

• In-depth protein characterization of 35 different international barley cultivars.
• Selection of eight cultivars to produce a new barley gluten reference material.
• The prolamin/glutelin ratio is 1.6 on average for barley.
• B/γ-hordeins are found in the prolamin and glutelin fraction.

## Graphical abstract

**Figure:**
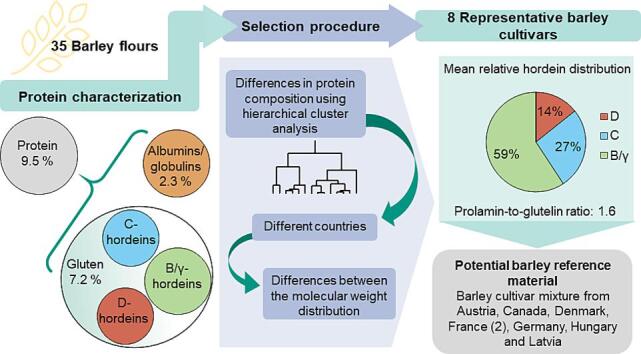

## 1 Introduction

Around 1% of the worldwide population suffers from celiac disease (CD) and 0.6 to 6% are estimated to suffer from non-celiac wheat sensitivity (Serena et al., 2020). CD patients commonly experience gastrointestinal symptoms such as chronic diarrhea, bloating and abdominal pain, but may also show various extraintestinal manifestations after consuming gluten (Green et al., 2015). Gluten intake can trigger autoimmune reactions in CD patients that lead to degradation of the villi in the upper small intestine, which results in malabsorption and nutrient deficiencies, if not treated. The only treatment so far is a strict gluten-free diet. For this reason, affected persons are dependent on gluten-free labeled foods (Choung et al., 2017).

Gluten is a complex storage protein mixture located in the endosperm of grains such as wheat, rye, and barley or their crossbred varieties. It is typically divided into the predominantly monomeric prolamins soluble in aqueous alcohols and polymeric non-soluble glutelins. The prolamins and glutelins of wheat are termed gliadins and glutenins. In rye or barley these fractions are called secalins and secalinins or hordeins and hordenins, respectively. The terms secalins and hordeins are more common and mainly used for barley and rye gluten protein types, because the separation into prolamins and glutelins according to solubility is inappropriate for rye (Xhaferaj et al., 2023) and barley. Barley gluten can further be classified into the monomeric C- and γ-hordeins and the polymeric B- and d-hordeins (Schalk et al., 2017).

Barley is commonly used for animal feed and it is the most important raw material for the malt and brewing industries. Gluten-free products, e.g., beers based on barley treated in appropriate ways to remove gluten, must not contain>20 mg of gluten per kg of the product according to the Codex Alimentarius (Codex Alimentarius Commission). The analytical requirements for gluten-free labeling are also stated in the Codex. The analytical method should be based on an immunological assay with a specific antibody reactive to CD-active gluten epitopes. In particular, the enzyme-linked immunosorbent assay (ELISA) based on the R5 monoclonal antibody reacting to specific epitopes within gluten proteins has been recognized as type I method (Codex Alimentarius Commission, Lacorn et al., 2019).

The epitope with the amino acid sequence QQPFP recognized by the R5 mAb is repetitively present in many CD-active peptides derived from gliadins, secalins, and hordeins. Other ELISAs such as those utilizing the G12 mAb are also acceptable with similar performance parameters. However, several studies have shown that different ELISA tests often give different results (Bugyi et al., 2013, Scherf, 2017, Amnuaycheewa et al., 2022, Xhaferaj et al., 2023). The main reasons are differences in extraction methods, standards (reference materials (RM)) used for calibration and specificity of the antibodies (Panda et al., 2017, Xhaferaj et al., 2020).

Different types of RM candidates have been proposed to quantitate gluten including recombinant proteins (García-García et al., 2020), isolated gluten protein fractions and types or flours (Huang et al., 2017, Schalk et al., 2017). Due to the considerable variability in gluten composition, gluten RM development encounters several difficulties. The gluten composition is influenced by genetics and environmental factors such as climatic conditions, fertilization and country of origin which cannot be eliminated (Hajas et al., 2018). Recent RM developments showed that mixing flours of different cultivars significantly reduced the effect of genetic and environmental factors (Schall et al., 2020). The currently used RM to calibrate many ELISA test kits is the so-called Prolamin Working Group (PWG)-gliadin, which has been isolated from a mix of 28 different European wheat cultivars. It is composed of wheat prolamins and is the best characterized RM available for gluten analysis so far (van Eckert et al., 2006).

However, the use of PWG-gliadin as calibrator in the R5 ELISA kit resulted in an average 5-fold (519%) overestimation of gluten in rye flours (Xhaferaj et al., 2023). One reason is that most ELISA methods mainly target prolamins and calculate the gluten content by multiplying the prolamin content by two. However, the assumption that prolamins and glutelins are present in equal proportions (prolamin/glutelin ratio of 1) does not always fit. Several studies have shown that this conversion factor for determining gluten is inappropriate (Wieser & Koehler, 2009). Especially for rye the conversion factor led to an overestimation of gluten in 32 rye cultivars that had a mean prolamin/glutelin ratio of 4.4 (Xhaferaj et al., 2023). Another reason for the overestimation of gluten is the specificity of the antibodies. The R5 mAb was raised against secalins (Osman et al., 2001), resulting in higher reactivity towards secalins.

The prolamin and glutelin content varies strongly depending on cereal species, cultivars, environmental conditions and processing from raw material to final product (Luo et al., 2019). Most studies focus on wheat gluten composition for use as a RM whereas little research has been done on other cereal proteins such as hordeins from barley. To date, there is no barley RM available for gluten quantitation. A reliable picture of the barley protein variability of a larger collection of different international barley cultivars has also never been reported so far.

To fill this gap, the aim of this study was to select specific barley cultivars with a potential as a new RM candidate for ELISAs and other analytical methods. Based on previous studies on RM development the hypothesis is that a mixture of different barley cultivars is more suitable compared to a single cultivar, because mixing reduces the genetic and environmental variability of gluten composition (Hajas et al., 2018, Schall et al., 2020). To be as representative as possible, an in-depth characterization of 35 barley cultivars from various countries was the focus of our study.

The leading countries (2021) in barley production are Russia (17.9 million tons), Australia (14.6 million tons), France (11.3 million tons), Germany (10.4 million tons) and Ukraine (9.4 million tons). The grain selection in the present study represents seven countries with a total production of 34.7 million tons of barley in 2021. Further, the selection of eight suitable cultivars representing the variability of barley protein composition will be shown. A blend of these eight cultivars will be used as a basis for the new RM. The new barley RM will help to improve the accurate quantitation of gluten to ensure food safety for CD patients.

## 2 Materials and methods

### 2.1 Sourcing of barley grains and flour preparation

Thirty-five barley samples were collected from seven different countries and geographical origins (Table S1). Kernels were milled to wholemeal flours on a laboratory mill (Cyclotec Mill 1093, Foss Tecator AB, Höganäs, Sweden). The mill was cleaned mechanically and with compressed air after each sample and the first 10 g of newly milled sample were discarded. Wholemeal flours were stored in zip-lock bags at 22 °C until further use. To obtain the mixture of the eight selected barley samples (PIX_FRA20, GKJ_HUN17, COC_FRA20, EVE_DEN20, JAK_GER20, CEL_CAN19, EVE_AUS20, KOR_LAT19), 500 mg of each flour were mixed and homogenized for 24 h in an overhead shaker.

### 2.2 Flour characterization

The moisture content of the flours was determined by the oven-drying method in duplicates according to ICC Standard No. 109/1. Crude fat content of the flours was analyzed in duplicates according to ICC Standard No. 136 using a Soxtec System HT-1043 instrument (Foss Tecator AB, Höganäs, Sweden). The crude protein content of the flours (nitrogen × 5.7) was determined by the Dumas method using a Leco FP 528 nitrogen analyser (Leco Corporation, St. Joseph, USA) in duplicates following ICC Standard No. 167.

### 2.3 Barley protein characterization

#### 2.3.1 Extraction procedure

The barley proteins were extracted according to the modified Osborne fractionation procedure (Wieser et al., 1998). The stepwise extraction (100 mg flour), magnetic stirring, vortexing and centrifugation was done exactly according to Xhaferaj et al. (2023). The extracted solutions of albumins and globulins (ALGL), prolamins and glutelins were diluted to 2 ml with the respective extraction solvents and filtered (0.45 µm Whatman SPARTAN, Cytiva Europe GmbH, Freiburg im Breisgau, Germany). The prolamin fraction was additionally reduced with 1% (w/v) dithiothreitol (DTT) resulting in the reduced (red.) prolamin fraction. The solutions were used for the chromatographic analysis of the proteins.

#### 2.3.2 Protein characterization by RP-HPLC

**Fig. 1:**
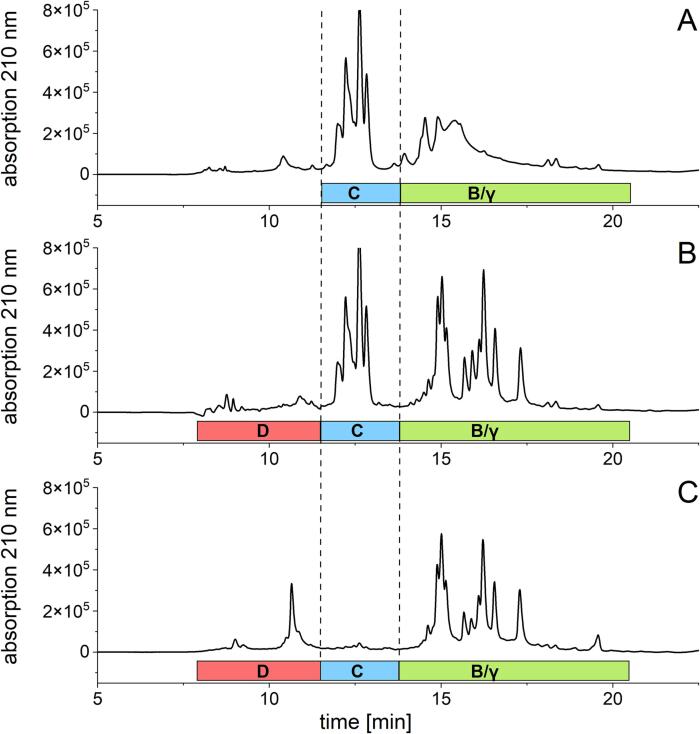
**RP-HPLC profiles of the Osborne fractions of the barley cultivar Pixel.** A: Unreduced prolamins, B: Reduced prolamins, C: Glutelins. With the barley protein fractions C: C-hordeins; D: d-hordeins and B/γ: B/γ-hordeins.

Protein content and distribution were determined using reversed-phase high-performance liquid chromatography (RP-HPLC). The instrument, column, mobile phase and the specific gradients applied for the separation were exactly as reported by Xhaferaj et al. (2023). Proteins were detected at 210 nm and quantitated using the corresponding absorbance areas of PWG-gliadin (van Eckert et al., 2006). The gluten content was calculated from the sum of red. prolamins and glutelins. The individual hordein types were quantitated based on their percentage of the total peak area. The evaluation of the chromatographic profiles and the classification of the hordein types (Fig. 1) was based on the literature (Gessendorfer et al., 2009, Schalk et al., 2017, Šimić et al., 2007).

#### 2.3.3 Relative molecular mass distribution by GP-HPLC

The relative molecular mass (M_r_) distribution of hordeins was determined using gel permeation HPLC (GP-HPLC). The instrument, column and mobile phase used for the separation were exactly as reported by Xhaferaj et al. (2023). Detection was performed using a DAD at 210 nm. Proteins with known M_r_ were used to determine the integration limits for specific M_r_ ranges. The proteins used were cytochrome *C* from horse heart (12.4 kDa), carbonic anhydrase from bovine erythrocytes (29 kDa) and albumin from bovine serum (66 kDa). The M_r_ ranges were categorized according to their molecular masses into the following fractions: 1: >66 kDa; 2: 66–29 kDa; 3: 29–12.4 kDa; 4: <12.4 kDa. In each section, the area under the curve was integrated and calculated as a percentage of the total area.

#### 2.3.4 SDS-PAGE

The proteins of the selected barley flours (20 mg) were extracted and separated according the protocol from Xhaferaj et al. (2023). The barley flour mixture of the eight selected cultivars was prepared as follows: The flour mixture (20 mg) and each cultivar (20 mg) were extracted. A NuPAGE 4–12% Bis-Tris protein gradient gel (1.0 mm, 10-well, Invitrogen, Carlsbad, CA, USA) and MOPS running buffer (50 mmol/L MOPS, 50 mmol/L Tris, 3.5 mmol/L SDS, 1 mmol/L EDTA, pH 7.7) were used. Prior to use, DTT (5 mmol/L) was added to the buffer as reducing agent (Lagrain et al., 2012, Geisslitz et al., 2020). The samples (PIX_FRA20, GKJ_HUN17 and COC_FRA20: 10 µl; EVE_DEN20, JAK_GER20, CEL_CAN19, and EVE_AUS20: 8 µl; KOR_LAT19: 5 µl) and the marker (5 µl) (PageRuler Unstained Protein Ladder (Thermo Scientific, Bremen, Germany) covering a range of 10 kDa to 200 kDa with 14 proteins) were loaded into the wells. The preparation of the gels (fixing, staining, destaining) and the instrument parameters used were applied exactly according to Xhaferaj et al. (2023). The gels were scanned with a Gel Doc EZ Imager (BioRad, Feldkirchen, Germany) and the M_r_ of bands were estimated based on the marker proteins by the AIDA Image Analysis software.

### 2.4 Gluten quantitation by ELISA

Two commercially available ELISA test kits were used for gluten quantitation: RIDASCREEN Gliadin Assay (limit of detection (LOD): 0.5 mg/kg of gliadin, limit of quantitation (LOQ): 2.5 mg/kg and gluten content calculation: gliadin content × 2) (R7001, R-Biopharm, Darmstadt, Germany) and AgraQuant Gluten G12 Assay (LOD: 2.0 mg/kg of gluten, LOQ: 4.0 mg/kg and gluten content calculation directly from the calibration) (COKAL0200, Romer Labs, Tulln, Austria). ELISA procedures were carried out according to the kit instructions. These two kits apply different monoclonal antibodies (R5 and G12, respectively) and different calibrators (PWG-gliadin and wheat gluten extract, respectively). To obtain a concentration in the calibration range, the barley flour extracts were additionally diluted 10.000-fold. The absorbances were determined using a microplate reader (iMarkTM Microplate Absorbance Reader, Bio-Rad, Hercules, CA, USA). The gluten concentrations were calculated from the absorbance values by the Bio-Rad Microplate Manager 6 software (Bio-Rad) using the curve fit and calculations suggested by the test kit manufacturer.

### 2.5 Statistics

For all quantitative values, means (n = 3) and absolute standard deviations (SD) were calculated. The Pearson correlation coefficients (r) were defined as r ≤ ± 0.54 no correlation, ± 0.54 < r ≤ ± 0.67 weak correlation, ± 0.67 < r ≤ ± 0.78 medium correlation, and r > ± 0.78 strong correlation (Thanhaeuser et al., 2014). All given correlations had a significance of p < 0.05. Additionally, one-way ANOVA (Tukey’s post hoc test, p < 0.05) was used to analyse the differences among the means of the M_r_ distribution (GP-HPLC) and RP-HPLC data. A hierarchical cluster analysis was carried out to find differences and similarities among barley cultivars and classify the different barley cultivars into groups. All statistical analysis was performed with the use of Origin 2021b software (OriginLab Cooperation, Northampton, MA, USA).

## 3 Results and discussion

### 3.1 Moisture, fat and protein content

**Fig. 2:**
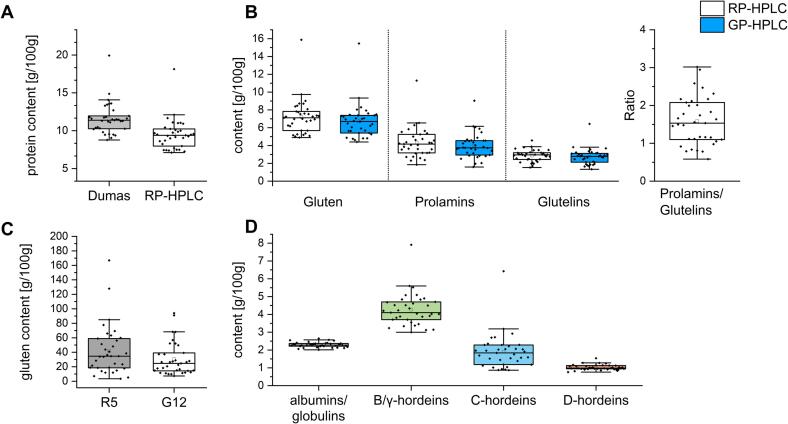
**Boxplots showing the protein characterization of 35 barley cultivars.** The box represents the 25th and 75th percentiles. The diamonds are the data points for each cultivar (n = 35). The small square in the box indicates the mean, the line the median. The whiskers indicate the upper (75th percentile) and lower (25th percentile) inner fence with a 1.5 interquartile range (whisker length determined by the outermost data point that falls within upper and lower inner fence). A: Protein content measured with Dumas and RP-HPLC. B: Gluten, prolamins, glutelins and the prolamin/glutelin ratio measured with RP-HPLC and GP-HPLC. C: Gluten content measured with R5 and G12 ELISA. D: Protein fractions measured with RP-HPLC.

The moisture content of the 35 barley samples ranged from 8.8 to 11.4% with a mean moisture of 10.1 ± 0.8%. The samples had an average fat content of 1.7 ± 0.3% (Tables S2 and S3). Fig. 2 in combination with Tables S3 to S5 provides the results of the in-depth characterization of the 35 barley cultivars. The average protein content measured by Dumas was 11.6 ± 2.0 g/100 g and higher compared to the RP-HPLC results (9.6 ± 2.0 g/100 g) (Fig. 2A). The protein content analysed by RP-HPLC was calculated using the sum of ALGL, red. prolamins and glutelins. The protein content of the samples ranged from 8.8 to 19.9% by Dumas and from 7.0 to 18.3% by RP-HPLC and the results of both methods correlated positively (r = 0.98). These results are comparable to those previously reported for barley where crude protein content ranged from 7.7% to 15.1% (Yu et al., 2017, Schalk et al., 2017). The sample KOR_LAT19 had a significantly higher protein content of 18.3 g/100 g compared to the other samples (7.1 to 12.1 g/100 g) which is why the results are presented separately in the following.

### 3.2 Gluten quantitation using RP-HPLC

The mean gluten content (sum of red. prolamins and glutelins) obtained by RP-HPLC was 7.2 ± 2.0 g/100 g and ranged from 4.7 to 9.3 g/100 g (15.9 g/100 g for KOR_LAT19) (Tables S2 and S3). When comparing the gluten content, barley showed a higher content than rye flours (mean of 32 rye samples: 4.3 g/100 g) (Xhaferaj et al., 2023). The hordein distribution was determined by integration of the corresponding fractions as shown in Fig. 1. The elution profiles and the integration ranges used for the quantitation of prolamin and glutelin fractions as well as the hordein types were comparable to the profiles reported by (Schalk et al., 2017). The elution profile does not show a clear separation of B- and γ-hordeins in the prolamin fraction (Fig. 1A). By reducing the prolamins a clearer separation was observed (Fig. 1B and 1C) for the B- and γ-hordeins. Since we do not know the exact composition of the individual peaks, both fractions were considered as one (B/γ-hordeins). The hordein types identified in the red. prolamin fraction were summed with those of the glutelin fraction, because D- and B/γ-hordeins were present in both fractions (Fig. 1). Based on the integration ranges (Fig. 1), the protein content of the 35 barley cultivars ranged from 2.0 to 2.7 g/100 g for ALGL, 0.8–1.5 g/100 g for d-hordeins, 0.4–2.3 g/100 g for C-hordeins, and 3.0–7.9 g/100 g for B/γ-hordeins (Fig. 2D, Tables S3 and S3). The relative protein distribution was on average 25% ALGL, 11% d-hordeins, 19% C-hordeins and 45% B/γ-hordeins. The dominant proteins within the hordeins were thus the B/γ-hordeins followed by the C-hordeins and the d-hordeins. This distribution pattern is in accordance with previous studies on hordeins (Gessendorfer et al., 2009, Schalk et al., 2017).

### 3.3 Gluten quantitation and relative molecular mass distribution by GP-HPLC

The gluten content analyzed by GP-HPLC ranged from 4.4 to 9.3 g/100 g of flour (15.5 g/100 g for KOR_LAT19) (Table S2). A positive correlation (r = 0.98) was found between the GP-HPLC and RP-HPLC results. The chromatograms were subdivided in four ranges to determine the M_r_ distribution: (1) > 66 kDa; (2) 66–29 kDa; (3) 29–12.4 kDa; (4) < 12.4 kDa (Table S5). Considering the mean values, the prolamins showed a distribution of 29.6% (1), 7.5% (2), 24.5% (3) and 38.4% (4). The sample PIX_FRA20 stood out significantly with a lower percentage of 15.6% (1) and the highest percentage of 55.1% (4) within the prolamins. The M_r_ distribution of the prolamin fraction changed after reduction with DTT to 2.3% (1), 3.3% (2), 35.4% (3) and 59.1%(4). There was a decrease of the highest M_r_ fraction (1) whereas the fractions (3) and (4) increased, due to the reduction of intermolecular disulphide bonds. Among the red. prolamins, the percentage of fraction (4) was comparatively high for all samples originating from Denmark, ranging from 71.3 to 80.1% (Table S5). The M_r_ distribution in the glutelin fractions resulted in the following average values: 11.4% (1), 8.3% (2), 29.7% (3) and 50.6% (4). Again, the values of fraction (4) of the samples from Denmark were high in comparison, resulting in lower percentages for the fractions (1), (2) and (3). The differences in the M_r_ distribution were used as one selection criterion (section 3.6).

### 3.4 Gluten quantitation with ELISA

In this study, the commonly used R5 sandwich ELISA according to Méndez and the G12 sandwich ELISA were used for gluten quantitation (Méndez et al., 2005, Morón et al., 2008). The gluten content ranged from 3.4 to 166.8 g/100 g using the R5 ELISA. The range of the gluten content determined by the G12 ELISA was narrower with 7.3 to 94.0 g/100 g (Fig. 2C). The gluten content was overestimated for most of the samples compared to the RP-HPLC results, except for GKJ_HUN17, MVI_HUN17 and MOR_HUN17 with ELISA recoveries of 59.7%, 93.5% and 68.2% compared to RP-HPLC, respectively, using the R5 ELISA. There was no correlation between the results of different kits (r = 0.52) and between the ELISA and RP-HPLC results (R5, r = 0.53; G12, r = 0.42). The results show that the different ELISA methods in our experiment did not give the same result. This can be attributed, e.g., to the different specificities of the antibodies and the use of an RM that is unsuitable for barley gluten, as reported in previous studies (Lexhaller et al., 2016, Scherf, 2017, Yu et al., 2017, Amnuaycheewa et al., 2022, Xhaferaj et al., 2023).

### 3.5 Conversion factor for barley gluten content estimation

According to the Codex, gluten is calculated by duplication of the prolamin content, based on the assumption that the prolamin/glutelin ratio is 1 (Codex Alimentarius Commission). In previous studies of protein distribution, different prolamin/glutelin ratios were found for different grain species and cultivars (Wieser and Koehler 2009). Overall, the prolamin/glutelin ratio of the 35 barley samples ranged from 0.6 to 3.0 depending on the cultivar, with an average of 1.6 ± 0.6 (Table S2). When comparing the RP-HPLC results with the ELISA results, there was an overestimation of gluten with the ELISA (Fig. 2B and 2C). Deviations of the prolamin/glutelin ratio from the usual assumed factor of 1 (conversion factor of 2) can lead to an under- or overestimation of gluten by ELISA test kits. Considering the prolamin/glutelin ratio of 1.6, the barley-specific conversion factor is calculated to be 1.6 instead of 2. Using this factor, the overestimation of barley gluten reduced the mean ELISA values for R5 from 41.9 to 34.0 g/100 g of gluten and for G12 from 30.7 to 24.9 g/100 g of gluten. However, compared to RP-HPLC, the values are still higher due to differences in reactivity of the R5 and G12 antibodies to barley gluten.

The protein fractionation into prolamins and glutelins based only on solubility is therefore less applicable for hordeins. Fig. 1 shows that there is no clear separation of B/γ-hordeins between the red. prolamins and glutelins. Similar results were observed for rye gluten (Xhaferaj et al., 2023). Besides the use of wheat-based protein isolates (PWG-gliadin) for ELISA calibration, differences in antibody specificity play an important role in the overestimation of gluten in barley and rye contaminated foods (Wieser & Koehler, 2009). Changing the conversion factor alone may result in more accurate quantitation of gluten by ELISA, but there is more to consider.

Further research was done on different hordein types and their reactivity with the R5 mAb in a sandwich ELISA test. C-hordein was found to be 10 to 20 times more reactive than the PWG-gliadin standard. In comparison, the gliadin standard was found to be 8 to 25 times more reactive than B-hordeins (Huang et al., 2017). In a separate study, the reactivities of prolamin and glutelin fractions from rye, barley, and wheat were compared using five different ELISA test systems. The findings suggested that barley prolamins showed a higher reactivity than wheat prolamins, while barley glutelins showed a lower reactivity (Lexhaller et al., 2016).

A barley-based RM with a known protein distribution in combination with a suitable conversion factor also taking antibody specificity into account may improve the quantitation of gluten.

### 3.6 Barley cultivar selection for reference material development

The selection of the relevant cultivars for RM production was based on qualitative and quantitative criteria and focused on differences in cultivar characteristics. The first selection criterion were typical RP-HPLC (Fig. 1) and GP-HPLC elution profiles. The visual examination resulted in the consideration of all 35 samples for further selection for representative barley cultivars, because all samples showed regular chromatographic elution profiles in RP- (Fig. 1) and GP-HPLC, as described previously (Šimić et al., 2007, Gessendorfer et al., 2009, Schalk et al., 2017, Huang et al., 2017). Slight differences in the peak heights were due to the different protein composition of the samples (e.g., the d-hordein peak range in the glutelin fraction). Overall, sample KOR_LAT19 stood out due to its high protein content (18.3%), which was also confirmed in our analysis of another sample from the current harvest year 2022 (data not shown). Despite the relatively high protein content, KOR_LAT19 was included in the following cultivar selection, because the purpose of the cultivar selection is to obtain a representative sample set, taking into account large differences as well.

The further selection process focused on the similarities and differences of the cultivar characteristics using quantitative data such as protein composition, gluten content and ELISA response (Table S3 to S5). To capture the variability, hierarchical cluster analysis was performed. This statistical tool defines clusters indicating differences between and similarities within the clusters and it resulted in five clusters (Table 1). Cluster C1 contained 20 different cultivars from seven countries including Austria (2), Canada (2), Denmark (4), France (5), Germany (3), Hungary (2) and Latvia (2). C1 contained all cultivars from France, which is the result of similarities in composition. The second cluster (*C*2) contained five cultivars (Canada (2), Germany (1) and Hungary (2)). Cluster C3 contained four cultivars, three of which were from Austria and one from Germany. The fourth cluster (C4) included four cultivars from Latvia (3) and Canada (1). Two cultivars (one each from Hungary and Canada) were present in cluster C5.

**Table 1:** **Content of protein, gluten, protein fractions and hordein types of the selected barley cultivars measured by RP-HPLC**.

Sample	Protein^a^	Gluten^b^	Prolamins	Glutelins	Albumins/Globulins	d-hordeins	C-hordeins	B/γ-hordeins	PROL/GLUT ratio^c^
	g/100 g								
PIX_FRA20	7.42^A^	5.02^G^	1.85^F^	3.17^C^	2.40^A^	1.03^C^	0.87^H^	3.12^E^	0.6
GKJ_HUN17	7.70^B^	5.69^F^	3.61^E^	2.08^F^	2.01^D^	0.95^C^	1.18^G^	3.55^D^	1.7
COC_FRA20	8.62^C^	6.25^E^	4.28^CD^	1.97^F^	2.37^AB^	0.97^C^	1.57^F^	3.71^D^	2.2
EVE_DEN20	9.04^D^	6.80^D^	3.65^E^	3.15^C^	2.24^C^	1.02^C^	1.76^E^	4.02^C^	1.2
JAK_GER20	10.00^F^	7.54^C^	4.05^D^	3.49^B^	2.46^A^	1.25^B^	2.06^D^	4.23^C^	1.2
CEL_CAN19	9.56^E^	7.32^C^	4.57^C^	2.75^D^	2.24^C^	1.02^C^	2.28^C^	4.02^C^	1.7
EVE_AUS20	11.10^G^	8.72^B^	6.28^B^	2.43^E^	2.38^AB^	0.97^C^	2.92^B^	4.83^B^	2.6
KOR_LAT19	18.14^G^	15.87^A^	11.29^A^	4.57^A^	2.27^BC^	1.54^A^	6.42^A^	7.91^A^	2.5
Mean	10.20	7.90	4.95	2.94	2.30	1.09	2.38	4.42	1.7

The values are given as means (n = 3), (g/100 g) and different capital letters indicate significant differences between the samples in each column (one-way ANOVA, Tukey’s post hoc test, p < 0.05).

a Sum of reduced prolamins, glutelins, albumins and globulins measured by RP-HPLC.

b Sum of reduced prolamins and glutelins measured by RP-HPLC.

c Ratio of reduced prolamins and glutelins measured by RP-HPLC.

The country of origin and the differences between the M_r_ distributions were further used as selection criteria for representative samples. At least one sample was selected from each cluster and each country, whereas three samples (GKJ_HUN17, EVE_DEN20 and COC_FRA20) were selected from C1, as it is the cluster containing most cultivars. Furthermore, JAK_GER20 (*C*2), EVE_AUS20 (C3), KOR_LAT19 (C4) and CEL_CAN19 (C5) were selected. Sample PIX_FRA20 was chosen additionally, because of the significant difference shown in the M_r_ distribution of the prolamin and glutelin fractions compared to the others, resulting in eight cultivars selected as representative cultivars for RM production (Table1).

### 3.7 In-depth characterization of the eight selected cultivars

#### 3.7.1 Protein content and gluten composition

The protein content of the eight selected samples measured with RP-HPLC ranged from 7.4 g/100 g (PIX_FRA20) to 18.4 g/100 g (KOR_LAT19) (Table 1). KOR_LAT19 had the highest gluten content with 15.9 g/100 g, followed by EVE_AUS20 with 8.7 g/100 g. The ALGL content was similar across all samples and ranged from 2.0 to 2.5 g/100 g. The results showed a strong correlation between the protein and gluten content (r = 0.96) which is in accordance with the results considering all 35 cultivars. The prolamin content was higher compared to the glutelin content for all samples except PIX_FRA20, which is reflected in the prolamin/glutelin ratio of 0.6 for PIX_FRA20 and above 1 for the others (Table 1).

**Fig. 3:**
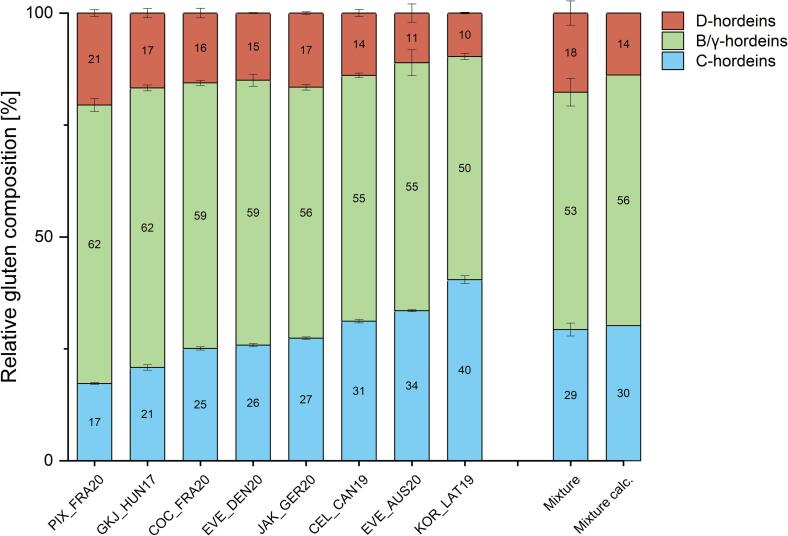
**Relative gluten composition of selected barley cultivars and their mixture.** The gluten composition was determined with RP-HPLC. The mixture consists of the flours of the selected 8 varieties in equal proportions. Mixture calc. is the calculated composition resulting from the mean values. Error bars indicate the standard deviations (n = 3).

The relative gluten composition of the selected samples was in agreement with the distribution of the 35 samples following the distribution pattern B/γ-hordeins > C-hordeins > d-hordeins (Fig. 3). Interestingly, PIX_FRA20 did not follow this rule because the d-hordein value (21%) of PIX_FRA20 was slightly higher than the C-hordein value (17%) and it showed the highest B/γ-hordein percentage overall (62%) (Fig. 3). KOR_LAT19 had the highest C-hordein percentage (40%), resulting in the lowest D- and B/γ-hordein percentages of 10% and 50%, respectively.

After mixing the eight selected flours in equal proportions (Fig. 3, mixture), the hordein distribution was as follows: 18% d-hordeins, 29% C-hordeins and 53% B/γ-hordeins. The calculated means of the mixture are shown in Fig. 3 (mixture calc.) as well. In comparison, mixture and mixture calc. both are very similar in hordein distribution, considering the error bars. Three samples (COC_FRA20, EVE_DEN20 and JAK_GER20) showed similar hordein distributions within and CEL_CAN19 had a hordein distribution comparable to the mixture calc. (Fig. 3). The selection shows a high variability, which was the basis of the selection process.

The comparable protein distribution of the mixture indicates that the chosen cultivars are indeed representative. The effect of genetic and environmental factors on gluten variability and ELISA response by analyzing wheat flours from multiple harvest years and a mixture has been examined (Schall et al., 2020). The study revealed that, in most cases, ELISA kits yielded higher gliadin recovery rates when using blended flour compared to individual cultivars. The harvest year did not have a significant impact on recovery values, but there were notable interactions between the ELISA kit, protein source, and harvest year. Mixing the flours reduced variability, thereby highlighting the benefits of using flour blends as a foundation for producing RM.

#### 3.7.2 SDS-PAGE

**Fig. 4:**
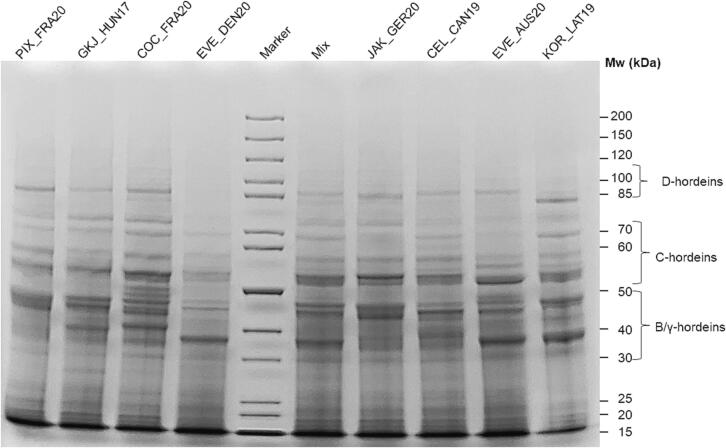
**SDS-PAGE of 8 selected barley flours and their mixture.** M: marker. 1: PIX_FRA20, 2: GKJ_HUN17, 3: COC_FRA20, 4: molecular weight marker, 5: Mix: barley flour mixture, 6: EVE_DEN20, 7: JAK_GER20, 8: CEL_CAN19, 9: EVE_AUS20, 10: KOR_LAT19, Mw: molecular weight.

Hordeins differ in their M_r_ and were separated by SDS-PAGE (Fig. 4). The M_r_ distribution of the barley flours and the mixture showed specific band ranges of 85–100 kDa (d-hordeins), 50–75 kDa (C-hordeins) and 30–50 kDa (B/γ-hordeins) (Schalk et al., 2018, Pont et al., 2020). All samples showed a typical hordein distribution on the SDS-PAGE gel (Molina-Cano et al., 2001). Light bands at around 85 kDa were visible for all samples indicating d-hordeins except for the comparably light band of KOR_LAT19. A comparably more prominent band right below 85 kDa can be observed for KOR_LAT19. In general, C-hordein and B/γ-hordein bands were more prominent for all samples. A more prominent band between 50 and 69 kDa can be observed for all samples indication the C-hordeins as well as a band below 60 kDa. In all samples two more prominent bands below 40 and below 50 kDa were visible indicating B/γ-hordeins. Sample COC_FRA20 showed several more clearly separated bands below 50 kDa in the B/γ-hordein range, which was not seen in the other samples. Three cultivars EVE_DEN20, EVE_AUS20 and KOR_LAT19 showed a more prominent band around 40 kDa. Comparing the distribution of the individual samples with the mixture, JAK_GER20 showed a very similar pattern which is in accordance to the relative gluten distribution (Fig. 3). The SDS-PAGE shows the apparent differences in M_r_ distributions which are due to protein polymorphisms between different cultivars (Echart-Almeida & Cavalli-Molina, 2000). Overall, the distributions are in accordance with previous studies (Gessendorfer et al., 2009, Tanner et al., 2013, Schalk et al., 2017, Pont et al., 2020).

### 3.8 Conclusion

The inconsistency of ELISA test systems when measuring gluten content using different assays such as R5 and G12 is a well-known issue. This variability is often due to differences in the antibody specificity of the test systems. Our experimental findings confirm the overestimation of gluten content when using the R5 and G12 mAb ELISA test systems on barley flours. The calibration standard also contributes to the overestimation. The use of PWG-gliadin for calibration, for example, is not suitable due to the differences between the proteins of wheat and barley. Additionally, we found that the typical separation of barley proteins into prolamin and glutelin fractions (Osborne fractions) may not be appropriate for hordeins. Because of that, the conversion factor for barley prolamins to gluten should be adjusted from 2 to 1.6. The current data highlights the importance of more suitable RM for barley gluten quantitation.

To address this issue and improve gluten quantitation, we characterized 35 different barley cultivars and used statistical methods and a selection procedure to identify eight cultivars that were representative and showed highest variability in terms of their protein composition. Mixing these selected cultivars aimed to reduce the environmental and genetic variability and provide a more appropriate gluten RM. The selected eight cultivars will be used in the following to prepare isolates in order to determine the reactivity and the suitability for inclusion in ELISA test systems. This paper discusses the challenges and presents an approach for improving gluten analysis to enhance the safety of food for individuals with CD. This paper additionally provides valuable insights into the characteristics of barley proteins as a whole using a high number of barley cultivars.

## Funding

Parts of the research reported here are related to the BME-EGA-02 - TKP2021 project supported by National Research, Development, and Innovation Fund of Hungary. Co-funded by the European Union (ERC, GLUTENOMICS, 101040437). Views and opinions expressed are however those of the author(s) only and do not necessarily reflect those of the European Union or the European Research Council Executive Agency. Neither the European Union nor the granting authority can be held responsible for them.

## CRediT authorship contribution statement

**Majlinda Xhaferaj:** Conceptualization, Data curation, Formal analysis, Investigation, Methodology, Visualization, Writing – original draft. **Gabriella Muskovics:** Data curation, Formal analysis, Investigation, Methodology, Writing – review & editing. **Eszter Schall:** Investigation, Methodology, Writing – review & editing. **Zsuzsanna Bugyi:** Conceptualization, Funding acquisition, Project administration, Resources, Supervision, Writing – review & editing. **Sándor Tömösközi:** Conceptualization, Funding acquisition, Project administration, Resources, Supervision, Writing – review & editing. **Katharina A. Scherf:** Conceptualization, Funding acquisition, Project administration, Resources, Supervision, Writing – review & editing.

## Declaration of Competing Interest

The authors declare that they have no known competing financial interests or personal relationships that could have appeared to influence the work reported in this paper.

## Data Availability

Data will be made available on request.
